# De-implementation of inequitable methadone policy and treatment: lessons learned from COVID-19 to support a more just methadone treatment system in the United States

**DOI:** 10.3389/fpubh.2026.1951510

**Published:** 2026-09-10

**Authors:** Kate Roberts, Andrew Goodman, Aaron Ferguson, Axl Kaminski, Tamika Spellman, Rachel Luba

**Affiliations:** 1 Disability Rights California, Sacramento, CA, United States; 2 Department of Psychiatry and Behavioral Science, University of California San Francisco, San Francisco, CA, United States; 3 Colorado Drug Policy Coalition, Denver, CO, United States; 4 National Survivors Union, Greensboro, NC, United States; 5 Department of Sociology, University of California Davis, Davis, CA, United States; 6 Grammy's Place, Birmingham, AL, United States; 7 Department of Psychiatry, Columbia University, New York, NY, United States

**Keywords:** de-implementation, low-value care, methadone, opioid use disorder, patient advocacy

## Abstract

Despite decades of evidence supporting methadone as a life-saving treatment for opioid use disorder, its delivery in the US has been stymied by stigmatizing dispensation policies that are neither evidence-based nor person-centered. Implementation failures and regulatory challenges have resulted in a significant treatment gap in the US and less than 30% of people with OUD enter and remain in treatment. In this article, we provide a historical overview of the methadone treatment (MT) system in the US and outline why it requires de-implementation. We present the COVID-19 public health emergency as a naturalistic experiment in de-implementation, outline evidence from this period on how COVID-19 related flexibilities influenced treatment outcomes and patient experiences and present a de-implementation framework to contextualize these changes while imagining further expansion of de-implementation efforts to support sustained change. We highlight how patient advocacy can shape further de-implementation while noting current threats to progress.

## Introduction

Methadone is a highly effective, lifesaving medication for opioid use disorder (MOUD), but its delivery in the United States (US) has been significantly hindered by stigmatizing policies and rules and regulations governing its delivery that are neither evidence-based nor person-centered. Decades of misalignment with critical components of effective healthcare has led to a notable treatment gap. Less than 30% of people with OUD enter treatment, and of those who do, the majority leave before reaching a stable dose (1, 2). In this article, we provide a historical overview of the methadone treatment (MT) system in the US and outline why it requires de-implementation. We define de-implementation as the process of discontinuing or terminating low-value practices that are potentially harmful and/or less effective than alternative practices (3). Low-value care can harm patients directly, cause indirect harm by requiring burdensome and/or unnecessary components of care, and reduce or threaten healthcare systems (4). We explore how COVID-19 functioned as a naturalistic opportunity for de-implementation, examine in detail what can be learned from this period, and imagine how patient advocacy can contribute to further de-implementation. We conclude with current threats and remaining barriers to a just, equitable, and person-centered MT system – one that can reach and retain people at risk of overdose, offer opportunities for lasting stability and engagement, and address past policy failures.

## Historical overview

Methadone is among the most effective evidence-based treatments for OUD, associated with substantial reductions in overdose and all-cause mortality, illicit opioid use, HIV and hepatitis C transmission (5–7). Yet the US regulates methadone unlike any other medication for a chronic condition, and unlike almost any other high-income country. Methadone for OUD is dispensed almost exclusively through federally certified Opioid Treatment Programs (OTPs) operating under 42 CFR Part 8 and overseen through a multilayered system involving SAMHSA, the Drug Enforcement Administration (DEA), accrediting bodies, and state opioid treatment authorities (SOTAs) (8). OTPs must comply with extensive federal and state requirements governing admission, observed dosing, urine drug testing, counseling, take-home eligibility, recordkeeping, and diversion control. By contrast, buprenorphine is prescribed in office-based settings by clinicians holding standard DEA registration (9, 10). This asymmetry is not based on evidence or clinical need but is historical in origin.

The OTP model inherited a regulatory posture toward opioid maintenance that predates methadone by half a century. In the largely unregulated medical marketplace of the late nineteenth century, physicians used opium, morphine, and heroin to treat a wide range of conditions; by 1888, 15% of prescriptions written in Boston were for opioids, and opioid prescribing was especially common among upper-middle-class women (11–13). Shifting professional norms, efforts to regulate the international opium trade, and xenophobic anxieties linking opium to Chinese immigrants converged to produce federal prohibition. The Harrison Narcotic Act of 1914, originally intended to regulate medical opium, was interpreted to bar opioid maintenance altogether, and Webb v. U.S. (1919) declared maintenance prescribing illegitimate. The approximately 44 municipal narcotic clinics operating between 1919 and 1921—which historians credit with reducing petty crime, easing court congestion, and stabilizing patients under physician supervision—were shuttered under Treasury Department pressure, and 25,000 physicians were indicted for Harrison Act violations between 1919 and 1935 (11, 14, 15). The result was a de facto abandonment of people with opioid dependence by medicine: patients moved from clinics into illicit markets, physicians were deterred from maintenance prescribing, and addiction treatment (namely abstinence-based and detoxification models) became siloed from standard medical practice or any evidence base (11, 14, 16).

Rising heroin use in the 1960s prompted renewed interest in treating opioid dependence with opioids. The trials conducted by Vincent Dole and Marie Nyswander established methadone's efficacy, with FDA approval in 1972. That approval coincided with the Nixon-era “War on Drugs” and political anxieties tying drug use to immigration, dissent, and social disorder (11, 14, 17, 18). Federal regulation followed immediately: the Comprehensive Drug Abuse Prevention and Control Act of 1970 separated MT from general medicine under dual FDA and DEA oversight, and the Narcotic Addict Treatment Act of 1974 established Narcotics Treatment Programs—later OTPs—as the sole authorized providers. (9, 17) Methadone was thus legitimized and quarantined in the same gesture.

Fifty years later, the MT system still bears the marks of its founding. Daily observed dosing, rigid take-home schedules, mandatory counseling, routine toxicology testing, and extensive surveillance derive not from evidence about methadone's safety or effectiveness but from efforts to control diversion and to regulate people who use drugs, particularly in racially marginalized and low-income communities (18–20). When oversight shifted from the FDA to SAMHSA with revisions to 42 CFR Part 8 in 2001, the compliance-oriented, surveillance-heavy architecture remained (21). Internationally, this model is the exception. Canada, Australia, and several European nations deliver methadone through pharmacies, primary care, and integrated community health systems, and these lower-threshold models maintain safety and retention while reducing treatment burden (22–24). Further, treatment gaps in these countries are correspondingly smaller: 70% of Canadians with OUD report past-year treatment access, and most EU nations exceed the World Health Organization's target of 40% MOUD coverage (25, 26).

Within the US, access varies sharply because states retain authority over OTP licensure, Medicaid reimbursement, telehealth, take-home dosing, and operations (27–29). The resulting inequities fall on rural communities, low-income patients, people with unstable housing, those under criminal-legal supervision, and racially marginalized groups (28, 30–33). In some regions, patients travel hours daily and even where clinics are dense, attendance requirements, restrictive hours, and rigid definitions of “stability” interfere with employment, caregiving, disability accommodation, and community life.

The distinction between methadone and the apparatus built to deliver it is the one this paper turns on. Methadone's evidence base has been settled for fifty years. The requirements structuring its delivery with daily attendance, observed dosing, mandatory counseling, routine toxicology testing, and restricted take-home access were not derived from an evidence base and have not acquired independent support since. What they reliably produce is burden – borne by patients through demands of compliance, and by a treatment system that prioritizes surveillance over care (4). These conditions represent low-value practices and highlight a need for de-implementation (3).

### COVID-19 as a naturalistic de-implementation

In many ways the public health emergency of COVID-19 functioned as a naturalistic de-implementation of some of the most problematic regulations and barriers within MT in the US. In March 2020, SAMHSA issued unprecedented temporary exemptions to 42 CFR Part 8 to reduce in-person visits while preserving treatment continuity (34, 35). These changes, the most substantial shift in methadone policy in decades, effectively suspended many longstanding regulatory assumptions underpinning OTPs. SAMHSA authorized up to 28 days of take-home methadone for “stable” patients and up to 14 days for “less stable” patients, expanded telehealth for counseling and evaluations, and granted OTPs more discretion in determining take-home eligibility and clinic operations (36–38). Surveillance-heavy practices previously seen as essential safeguards (e.g., frequent observed dosing and rigid in-person requirements) were rapidly loosened under emergency conditions. Importantly, COVID-era flexibilities did not de-implement MT itself but partially de-implemented restrictive delivery structures. This distinction is critical. Methadone remained a highly evidence-based intervention throughout the pandemic, while many regulatory practices were re-contextualized as modifiable rather than inherent components of care.

The pandemic therefore created a natural policy experiment allowing researchers, providers, and patients to observe the effects of removing or reducing treatment restrictions in real-world clinical settings. Emerging evidence from this period challenged assumption of the traditional OTP model and demonstrated improved patient autonomy, reduced treatment burden, greater flexibility in daily life, and increased satisfaction with care (19, 39–41). Patients described reduced transportation strain, improved ability to maintain employment and caregiving, and greater capacity to participate in community life when freed from daily clinic attendance requirements (19, 42). Importantly, many patients framed these changes not simply as increased convenience, but as a meaningful reduction in institutional control and surveillance. Prior qualitative research has described MT as “liquid handcuffs,” in which highly restrictive policies constrain autonomy and shape nearly every aspect of daily life (19, 20). Expanded take-home dosing disrupted some of these dynamics by allowing patients greater control over medication management and reducing the visibility of treatment participation within their social and occupational lives. From a health equity perspective, these changes were particularly meaningful for patients facing transportation barriers, unstable housing, caregiving responsibilities, disability-related access challenges, or employment constraints—groups historically burdened most heavily by rigid OTP attendance requirements.

Research from this period also challenged longstanding fears regarding widespread diversion and safety risks. Although concerns regarding overdose and diversion were the basis for highly restrictive methadone regulation, evidence suggests there were no major increases in methadone-related harms attributable to expanded take-home allowances (43–46). Several studies documented stable or improved treatment retention under these more flexible models, while systematic reviews found little evidence of diversion or increased risk predicted by opponents of reform (41, 42, 47–49). These findings directly challenged notions that highly restrictive attendance and surveillance practices were necessary to maintain safety or treatment effectiveness.

At the same time, COVID-era changes highlighted the limits and complexities of de-implementation within deeply institutionalized systems. Federal authorization alone did not guarantee practice change. Implementation of SAMHSA flexibilities varied substantially, revealing how organizational culture, institutional caution, reimbursement structures, and longstanding regulatory norms could constrain de-implementation even after formal policy changes. Many OTPs implemented only partial flexibilities or maintained restrictive practices despite new federal guidance, with some patients reporting that “nothing really changed” (50–53). Some states never adopted expanded take-home policies, while others later rescinded them. (38, 54).

These findings underscore a core insight from de-implementation science: formally changing policy may be insufficient to dismantle entrenched institutional practices. Regulatory flexibility may be necessary but not sufficient when provider beliefs, organizational incentives, reimbursement systems, liability concerns, and professional cultures maintain older models of care. MT during COVID-19 therefore illustrates both the possibilities and limitations of de-implementation within healthcare systems shaped by decades of criminalization, surveillance, and unsupported risk-management logics. Such findings also highlight the need for continued and expanded efforts to study and apply effective de-implementation strategies within MT, and to more effectively characterize clinic and systems-level factors that maintain low-value practices.

### Patient advocacy and re-imagining MT

COVID-19 also catalyzed patient advocacy and drug user organizing around methadone reform. Patients, advocates, and drug user unions advocated permanent transformations to MT based on COVID-era flexibilities. In many ways, COVID-19 exposed the possibility of de-implementing restrictive policies, while solidifying the extent to which patients viewed such practices as unnecessary and harmful. Patients began meeting regularly at the height of the COVID-19 pandemic as part of the *Urban Survivors Union's (USU) Methadone Monday* call. USU is a national group of directly impacted advocates for drug user health. Increased digital communication during the pandemic created new opportunities for patients to connect and laid the groundwork for envisioning a MT system that aligns with the real and stated needs of people who use drugs. The gap between what providers and OTPs assumed patients want or need, and the realities of drug use and treatment become evident as more patients organized and collaborated. These efforts culminated in a *Covid-19 Treatment Recommendations* sign on letter that was endorsed by hundreds of individuals and organizations, the *Methadone Manifesto*, and the nation's first patient-led conference for MT reform (*A Roadmap for Change)* (55–57). Thus emergency revision to an ineffective regulatory system and novel avenues for patient connection created an unprecedented opportunity for long held grievances and aspirations of MT patients to be heard, galvanizing a national cohort of patients toward reform. But to reimagine opioid treatment, it is necessary first to acknowledge the numerous ways that clinic policies violate basic autonomy and deter patients. Through many moments of shared anger and frustration emerged a desire to find concrete alternatives. A unifying thread among patient activists is the philosophy of “nothing about us without us”, a mantra that emerged as part of the disability rights movement and perfectly captures the essence of MT advocacy. To capitalize on the momentum of COVID-19 related changes, for MT to progress in patient-centered ways, and for effective de-implementation of low-value MT practices, those who are most impacted must hold formative influence. This will require significant investment on the part of healthcare systems, researchers, and providers to create avenues for actionable input while exploring ways to shift internal culture that stymie meaningful change. The current system has seen very little change for over 50 years, and this has solidified a status quo bias. For ongoing and meaningful de-implementation to continue, advisory initiatives must be built on meaningful inclusion, fair compensation and assurance that patient recommendations are actionable and enforced and a range of patient perspectives (both “compliant” and “noncompliant”) must be represented.

### Selecting a de-implementation framework for MT in the US

Selecting a de-implementation model may depend on whether the goal is to reduce, replace, restrict, or remove (without replacement) an existing approach (3). As there is no compelling evidence that MT should be removed, or that the current system should be applied in restricted settings, a combination of efforts to reduce and replace low-value policies is warranted (3). In envisioning more just MT in the US, focus on de-implementation frameworks (over pure implementation science frameworks) is supported by de-implementation's emphasis on behavior substitution, behavior monitoring, and restructuring of social environments to support change (58). The Choosing Wisely De-Implementation Framework (CWDIF) builds on theory-derived interventions that ask (1) What key players need to change their behavior and what needs to be done differently? (2) What barriers and facilitators must be addressed to support change? (3) What strategies could be used to surpass barriers and bolster facilitators? And (4) How will successful change be measured? (59) CWDIF is a helpful tool for imagining de-implementation of low-value MT practices. CWDIF is conceptualized across 5 phases, and in Table 1 we provide an example of how COVID-era changes can be contextualized under this framework, while highlighting remaining and ongoing work. We note that this forced de-implementation differs from how a truly patient-centered, patient-led de-implementation effort could have unfolded, and note what was left out in each phase of this naturalistic de-implementation.

**Table 1:** Contextualizing COVID-era changes within the choosing wisely de-implementation framework (CWDIF).

Phase 0	Phase 1	Phase 2	Phase 3	Phase 4
Identification of low-value healthcare	Identification of priorities for de-implementation	Identification of barriers to de-implementation and potential interventions to overcome barriers	Evaluation of de-implementation outcomes	Spread of effective de-implementation
**Status:** completed	**Status:** partially completed	**Status:** partially completed	**Status:** ongoing	**Status:** ongoing
Traditional MT models and daily clinic attendance pose significant public health risk during COVID-19. (35)	SAMHSA final rule updates increase flexibilities around take-home dosing, enable broader use of telehealth for intake and counseling visits, and grant OTP-level discretion on take-home dosing. (35, 62)	**Barriers:** •State-level discretion in adopting final rule flexibilities (28, 38, 54) •Institutional caution, organizational culture and stigma (50–52) •Reimbursement structures favoring in-person visits (63, 68, 69) •Continued separation of MT from other medical care (55) **Potential Interventions:** •Patient advocacy (20, 56, 57, 70) •Research evaluating COVID-19 era outcomes (19, 39–49)	**Available Evidence**: COVID-19 era flexibilities have been linked to stable treatment retention, no evidence of increased overdose or diversion, qualitative reports of increased patient satisfaction and autonomy. (19, 39–49)	**Barriers:** •High variability by state. (27, 38, 54) •Current threats to nullify portions of the 2024 Final Rule. (60) •Current efforts to de-legitimize MOUD as a long-term treatment. (62) •Financial incentives that favor in-person and frequent visits. (63, 68, 69) **Potential Interventions:** •Funding for technical assistance, education and training for OTPs to de-implement low-value MT practices •Investment in research to study and disseminate clinic and provider-level behavior changes to support de-implementation of low-value MT practices •Efforts to disseminate and provide guidance on how patients can advocate for de-implementation of low-value care and adoption of person-centered care •Continued efforts to explore alternative models of methadone delivery outside of OTPs •Efforts to standardize and implement policies for patients to file grievances when clinics fail to implement supported flexibilities •Critical analyses of OTP ownership and how revenue priorities may perpetuate low-value MT •Alignment of federal, state, and regulatory and state systems around de-implementation of low-value MT •Legislation to support new pathways for MT
**What was left out in COVID-19 era de-implementation?**				
Changes were motivated by a public health emergency rather than decades of patient advocacy for less burdensome, patient-centered care.	Patient-centered efforts to consider new models of MT in pharmacies and standard healthcare settings.	•Relaxing of federal regulations does not guarantee state and local-level adoption •The culture of cruelty and carceral nature of OTPs can remain even with increased flexibilities •Investment in efforts to provide compensated opportunities for patient advocates and affected patients to inform policy changes	•Efforts to define and measure outcomes that are most meaningful to people on methadone and people who use drugs •Efforts to measure and address clinic-level factors that perpetuate low-value care	

In considering the effective spread of de-implementation as we look to the future (Phase 4), we highlight what can be learned from patient advocacy and direct patient input. Healthcare systems and providers should clarify and make patients aware of differences in organizational policy and federal regulation and create clear paths for input on each. Organizational policy not dictated by federal regulation, should remain open to revision with patient input. When patient recommendations misalign with federal guidelines patients should have clear avenues for advocacy and a right to the substantiation of policy. This will require revisions to a significant number of existing rules and efforts to pilot, research, and substantiate rules prior to their implementation. Patients also need an improved process for grieving, and a clear expectation around their rights. Providers can establish ombuds departments that are adequately staffed and have actionable influence on policy, and who can communicate currently non-negotiable regulations in a patient-centered manner.

### Current threats and future directions

Gains made through COVID-era methadone flexibilities and the 2024 revisions to 42 CFR Part 8 remain politically and institutionally fragile. While these revisions were the most significant federal modernization of MT in decades, they did not fully dismantle the exceptional regulatory structure governing MT or guarantee more flexible, dignified, or accessible care. Several current threats could reverse or neutralize the progress made thus far.

The most direct threat is federal legislation that would nullify core portions of the 2024 final rule. H.R. 5629, introduced in the 119th Congress states that final rule “Medications for the Treatment of Opioid Use Disorder […] “shall have no force or effect” (H.R. 5629; except for the portion relating to OTP accreditation). (60) If enacted, this bill would undo several of the most important reforms and reinstate many of the barriers that COVID-era flexibilities safely relaxed.

Another threat lies in state-level resistance and uneven de-implementation. States retain authority over OTP licensing, Medicaid reimbursement, SOTA guidance, scope of practice, and operational requirements. Thus, even if the 2024 federal flexibilities were codified, they could be narrowed by state rules favoring restrictive models. During and after COVID-19, states varied widely in take-home dosing expansion, and some states that initially permitted it later opted out (54). Importantly, states that continued expanded take-home policies did not experience a detectable increase in methadone-related overdose deaths compared with states that opted out, suggesting state-level retrenchment may reflect political and institutional caution rather than public health necessity (54).

Organizational non-implementation must also be considered. Even when federal and state rules allow flexibility, individual OTPs can opt to maintain restrictive policies. The final rule gives OTPs discretion to determine take-home dosing within federal parameters and directs clinical decision-making toward individualized assessment, shared decision-making, overdose prevention, and patient circumstances (34, 61). This discretion can support patient-centered care when guided by harm reduction principles and patient input but can maintain the status quo when shaped by stigma, liability concerns or abstinence-only cultures of surveillance and compliance (36).

A fourth threat is the renewed political framing of long-term MOUD as undesirable. Evidence supports methadone and buprenorphine as lifesaving medications that are most beneficial when used as long-term treatment for a chronic condition (5, 7). Yet some political rhetoric portrays MOUD as “substituting one drug for another” or as acceptable only when oriented toward discontinuation. Recent SAMHSA guidance has emphasized holistic care models over “medication-only models” and calls for at least annual review of continued medication use, including possible tapering or discontinuation (62). Although periodic review can be clinically appropriate when patient-centered and voluntary, this language could be used in coercive or biased ways that favor abstinence-only models and place court or program expectations over patient goals.

Structural barriers that segregate MT from ordinary health care continue to cause problems. Certificate-of-need requirements, clinic caps, local opposition to OTPs (“Not in My Backyard” (NIMBY-ism)), limits on medication units, and exclusion of methadone from pharmacy and office-based delivery impose scarcity and burden (27). These restrictions are especially harmful in rural communities and for patients with limited transportation, unstable housing, disability, employment obligations, caregiving responsibilities, or criminal-legal supervision (23). Even with take-home rules becoming more flexible, patients cannot benefit from MT if there is no accessible point of treatment entry.

Finally, OTPs are increasingly owned by Private Equity (PE) firms, which have been linked to increased costs to patients and decreased quality of care (63, 64). Notably, PE firms own 30% of all OTPs in the US, one of the highest shares of ownership across healthcare sectors (65–67). Medicaid and Medicare reimbursement provide reliable revenue streams for PE firms and reimbursement and profits have historically been higher for clinics that bill for more services (68, 69). Thus, when patients attend more in-person visits and receive more billable services and more testing (e.g., urine drug screens), reimbursement increases. Focus on profit margins and revenue streams therefore creates a conflict of interest for PE firms and may foster opposition to de-implementation of low-value MT practices. Concentration of OTP ownership also increases the capacity for PE firms to lobby and influence regulation around MT, as was noted with an industry-backed effort to oppose legislation to reform methadone delivery in 2024 (66, 68).

## Conclusion

In this paper, we aimed to outline the historical context that enabled development of a flawed and persistent MT system in the US. We aimed to highlight how this historical context contributed to an inherently low-value system, and how COVID-19 created a naturalistic experiment in de-implementation that provides the foundation for further patient-led and patient-centered de-implementation efforts to replace low-value policies with compassionate, dignified, and evidence-based care. Notably, recently introduced legislation (H.R. 9790/ S.4941) seeks to modernize and expand access to MT by enabling dispensation by qualified practitioners in community pharmacies (70). Future intentional, patient-led de-implementation efforts have the capacity to address the opioid overdose crisis in the US (while declining, still a leading cause of death), to narrow the MOUD treatment gap, and to dramatically improves lives (1, 71). As noted, growing evidence suggests that the benefits of COVID-19 related flexibilities in MT far outweighed the risks. Nonetheless, adoption of these flexibilities has varied greatly between states, and new efforts to reverse the progress made thus far threaten sustainable change (54, 62). Further, deeply ingrained biases, and stigmatizing clinic cultures necessitate concerted de-implementation research that can inform clinic-level behavior change, behavior monitoring, and behavior modification that can support better care. Despite remaining challenges, there is reason to believe that patient and provider advocates galvanized by decades of low-value care, growing evidence that de-implementation is possible and produces favorable outcomes, and legislation to modernize opioid treatment will continue to help reimagine a more just future for MT.
